# Short-term cryopreservation and thawing have minimal effects on *Plasmodium falciparum ex vivo* invasion profile

**DOI:** 10.3389/fcimb.2022.997418

**Published:** 2022-09-20

**Authors:** Laty G. Thiam, Felix Ansah, Makhtar Niang, Gordon A. Awandare, Yaw Aniweh

**Affiliations:** ^1^ West African Centre for Cell Biology of Infectious Pathogens, College of Basic and Applied Sciences, University of Ghana, Accra, Ghana; ^2^ Department of Biochemistry Cell and Molecular Biology, College of Basic and Applied Sciences, University of Ghana, Accra, Ghana; ^3^ Pôle Immunophysiopathologie et Maladies Infectieuses, Institut Pasteur de Dakar, Dakar, Senegal

**Keywords:** *Plasmodium falciparum*, cryopreservation, thawing protocols, culture-adaptation, invasion phenotype

## Abstract

*Ex vivo* phenotyping of *P. falciparum* erythrocyte invasion diversity is important in the identification and down selection of potential malaria vaccine targets. However, due to the lack of appropriate laboratory facilities in remote areas of endemic countries, direct processing of *P. falciparum* clinical isolates is usually not feasible. Here, we investigated the combined effect of short-term cryopreservation and thawing processes on the *ex vivo* invasion phenotypes of *P. falciparum* isolates. *Ex-vivo* or *in vitro* invasion phenotyping assays were performed with *P. falciparum* clinical isolates prior to or following culture adaptation, respectively. All isolates were genotyped at Day 0 for parasite clonality. Subsequently, isolates that were successfully culture-adapted were genotyped again at Days 7, 15, 21, and 28-post adaptation. Invasion phenotyping assays were performed in isogenic isolates revived at different time points (3, 6, and 12 months) post-cryopreservation and the resulting data were compared to that from *ex-vivo* invasion data of matched isogenic parental isolates. We also show that short-term culture adaptation selects for parasite clonality and could be a driving force for variation in invasion phenotypes as compared to *ex vivo* data where almost all parasite clones of a given isolate are present. Interestingly, our data show little variation in the parasites’ invasion phenotype following short-term cryopreservation. Altogether, our data suggest that short-term cryopreservation of uncultured *P. falciparum* clinical isolates is a reliable mechanism for storing parasites for future use.

## Introduction

*Plasmodium falciparum* uses complex mechanisms for efficiently invading human erythrocytes and evading the host immune response (Wright and Rayner, 2014; Cowman et al., 2017), therefore, a better understanding of these mechanisms is critical for developing effective vaccines. Studies with *P. falciparum* field isolates are important for providing a clinically relevant elucidation of the molecular mechanisms involved in host-parasite interactions and pathogenesis of malaria (Ahouidi et al., 2016; Yap et al., 2019). One of the key approaches used in investigating molecular interactions at the host-parasite interface is phenotyping parasites for their erythrocyte invasion pathways. Invasion phenotyping generates data that are more representative of the parasite’s natural biology when these assays are conducted on parasites *ex vivo*, or during their first replicative cycle in culture. However, direct processing of clinical isolates requires advanced laboratory equipment and technical capacities, which are not always available in the clinics in the remote areas of endemic countries. Therefore, parasites isolates collected from patients at point-of-care usually need to be processed for cryopreservation, storage and transportation to laboratories with the requisite facilities for phenotyping (Ahouidi et al., 2016). These frozen parasites are then thawed using optimized protocols that minimize erythrocyte lysis and ensure maximal parasite survival. Therefore, it is important to determine the impact of any, of the cryopreservation and thawing processes on the parasites’ biology in general and their invasion phenotypes.

In the last decade, many laboratories reported on the invasion phenotypes of *P. falciparum* clinical isolates. However, cross-study comparison of these data is challenged by the use of different approaches to characterize such phenotypes (Ahouidi et al., 2016). The bulk of these phenotypic data were collected from cryopreserved parasites (Okoyeh et al., 1999; Lobo et al., 2004; Nery et al., 2006; Lopez-Perez et al., 2012) while only a few were from *P. falciparum* clinical isolates that were directly assayed after collection (Baum et al., 2003; Jennings et al., 2007; Lantos et al., 2009). Moreover, in some cases parasites were allowed to grow *in vitro* for at least five cycles prior to assay set up (Okoyeh et al., 1999; Lobo et al., 2004; Lopez-Perez et al., 2012), therefore, providing room for clonal selection. Unlike laboratory strains, *P. falciparum* clinical isolates are usually representative of a population of different clones, presenting intrinsic characteristics that could determine their *in vitro* adaptability. Given that different clones of the same isolate express distinct versions of surface antigens (Cortés, 2008), it could be that different clones use distinct invasion pathways and only the dominant clones of a given isolate will be reflected during invasion phenotyping experiments.

Alongside the cryopreservation, different laboratories may use different thawing protocols prior to culturing the parasites. Therefore, the consequences of variations in cryopreservation and thawing protocols in *P. falciparum in vitro* culture need to be assessed, especially with respect to parasite adaptation and subsequent invasiveness during *ex vivo* and *in vitro* phenotyping assays.

Previous literature has reported similar *ex vivo* adaptation rates in pre-cryopreserved isolates (Bowyer et al., 2015) as compared to fresh clinical isolates (Gomez-Escobar et al., 2010). However, these conclusions were drawn from studies using isolates of different isogenic backgrounds, which were phenotypically and functionally distinct. In this study, parasites from the same isogenic backgrounds were used to investigate the effect of freeze-thaw protocols on *P. falciparum* *ex vivo* invasion phenotypes and early *in vitro* adaptation.

## Materials and methods

### Sample collection and processing

*P. falciparum* clinical isolates were collected from 25 symptomatic children, aged 2 to 14 years old, visiting the LEKMA Hospital, in Accra between February 2017 and January 2018. LEKMA is a sub-urban area of Accra, the capital city of Ghana, a low malaria transmission setting, with an estimated entomological inoculation rate of <50 infective mosquito bites per person/year. The study was approved by the Institutional Review Board of the Noguchi Memorial Institute for Medical Research, University of Ghana (IRB00001276) and the Ghana Health Service Ethical Review Committee (GHC-ERC: 005/12/2017). All guidelines and principles contained in the approved protocol were duly followed in the execution of the project. A written informed consent was obtained from a parent and/or legal guardian and assent obtained for older children. Venous blood samples were collected in ACD vacutainers (BD Biosciences) and transported to the laboratory for processing within two hours after collection. At the laboratory, the infected erythrocytes were separated from the leucocytes through centrifugation at 2000 rpm and washed twice with RPMI1640 medium (Sigma). About 200µL of packed erythrocytes were put straight in culture, while the remaining sample was resuspended in glycerolyte in ~500 µL vials following the standard protocol (EVIMalaR, 2013) and stored in liquid nitrogen. Frozen vials were thawed at different time intervals using two distinct sodium chloride-based protocols. Vials were thawed using either a two-step protocol (12% NaCl and 1.6% NaCl) or a three-step protocol (12% NaCl, 1.8% NaCl and 0.9% NaCl supplemented with 0.2% Glucose) as per standard procedure (EVIMalaR, 2013). To minimize the effect of possible confounders, all reagents used in this study were prepared from single batches and stored as single-use aliquots.

### *Plasmodium falciparum in vitro* culture

**Figure 1:**
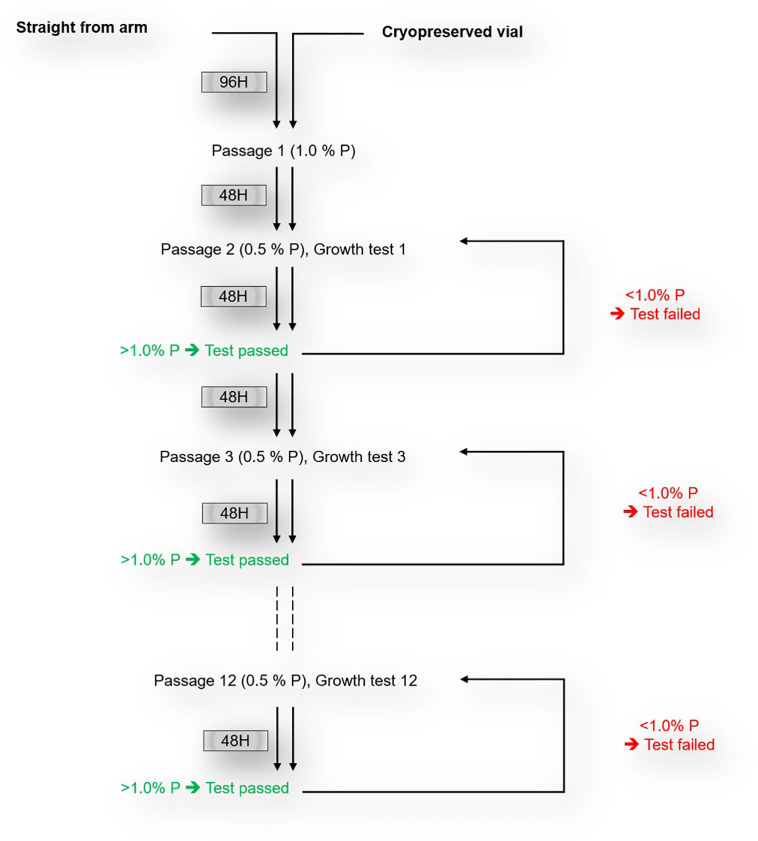
Schematics of *in vitro* culture-adaptation and growth test of freshly collected *P. falciparum* clinical isolates.

*P. falciparum* clinical isolates were cultured as per standard protocols (EVIMalaR, 2013). In brief, isolates were maintained at 37°C in RPMI1640 medium (Sigma), supplemented with 5% Albumax (Gibco), 2 mg/ml sodium bicarbonate, 50 µg/ml gentamycin (Sigma) and 2% AB^+^ heat-inactivated normal human serum (PAN Biotech, UK). All cultures were adjusted to 4% hematocrit using O^+^ erythrocytes from a single donor and incubated in an atmosphere of 2% O_2,_ 5% CO_2_ and balanced with Nitrogen. For isolates cultured upon arrival to the lab, the parasites multiplication rate (PMR), defined as the ratio of the parasitemia before and after re-invasion, was monitored for the first two *in vitro* cycles and fresh erythrocytes were only added after 96 hours in culture, while fresh erythrocytes were immediately added upon thawing of cryopreserved isolates. Following the addition of fresh erythrocytes, growth tests were performed to assess the PMR every 48 hours for the first three *in vitro* replicative cycles for the cryopreserved isolates and for up to 12 cycles for freshly culture adapted isolates. After each cycle, cultures were diluted to 0.5% parasitemia for the next cycle. Sample aliquots, taken following each replicative cycle were stained with 1 µM of Hoechst 33342 dye (Sigma Aldrich, UK) and the resulting parasitemia was assessed using flow cytometry. The growth test was considered successful only when the resulting PMR, measured after 48 hours, was greater than one (>1) (**Figure 1**). The median PMR of successful growth tests after twelve successive replicative cycles following the addition of fresh erythrocytes were considered as PMR of culture-adapted isolates.

### *Plasmodium falciparum* genotyping

*P. falciparum* genomic DNA (gDNA) was extracted from filter paper spots using the QIAamp DNA Blood Mini Kit (Qiagen, Hilden, Germany) and eluted in 30 µL elution buffer following the manufacturer’s instructions. The concentration and purity of the eluted gDNA were estimated using a NanoDrop One (Thermo Fisher Scientific, Madison, WI, USA). For each isolate, the presence of single or multiple parasite clones was assessed using a nested PCR approach as described earlier. The assays were performed using primers targeting the highly polymorphic regions of *msp1* (block 2) and *msp2* (block 3). All isolates that grew successfully during culture adaptation were genotyped after 7, 15, 21 and 28 days in culture and the number of clones was compared to that of the fresh isogenic isolate. The expected heterozygosity (H_E_) defined as the probability of being infected by at least two distinct alleles at a given locus was calculated as follows: H_E_ = [n/(n-1)] [(1-Σpi)], with n being the number of isolates and pi the allele frequency at a given locus (Diouf et al., 2019).

### Enzyme treatment and erythrocyte invasion phenotyping assays

Invasion phenotyping assays were performed using enzyme-treated erythrocytes (targets) from a single donor. Target erythrocytes were treated with either 250 mU/mL of neuraminidase, 1 mg/mL of trypsin or 1 mg/mL of chymotrypsin for 1 hour at 37° C with gentle shaking and washed thrice with RPMI1640 medium. The efficiency of enzyme treatment was assessed as previously described (Thiam et al., 2021). Treated erythrocytes were then labelled with 20 µM of carboxyfluorescein diacetate, succinimidyl ester (CFDA-SE) (Thermo Fisher Scientific) for two hours at 37° C with gentle shaking and protected from light exposure. For each isolate, schizont-infected erythrocytes were adjusted to 2% parasitemia and co-incubated with an equal volume of target erythrocytes in 96 well plates. All assays were performed in triplicates in a total volume of 100 µL at 2% haematocrit and incubated at 37° C for 24 hours. Parasitemia were adjusted to 1% for all isolates and invasion assays were considered successful only when invasion efficiency into control erythrocytes (untreated) was at least two-fold greater than the starting parasitemia. Plates were removed from the incubator and spun at 2,000 rpm for 3 minutes, after which the supernatant was discarded, replaced with a solution of 1 mM Hoechst 33342 to label the parasites’ DNA and incubated for an hour at 37° C. Plates were subsequently washed with 1X PBS and flow cytometry analyses were performed on a BD LSR Fortessa X-20 cytometer (BD Biosciences, Belgium). Invasion into target erythrocytes was determined by analysis of the proportion of Hoechst positive erythrocytes in 50,000 counted CFDA-SE positive cells. Percent invasion into enzyme-treated erythrocytes was expressed as a percentage of the invasion efficiency into labelled untreated erythrocytes.

## Results

### *P. falciparum* clinical isolates show different growth patterns during early *in vitro* culture adaptation

**Figure 2:**
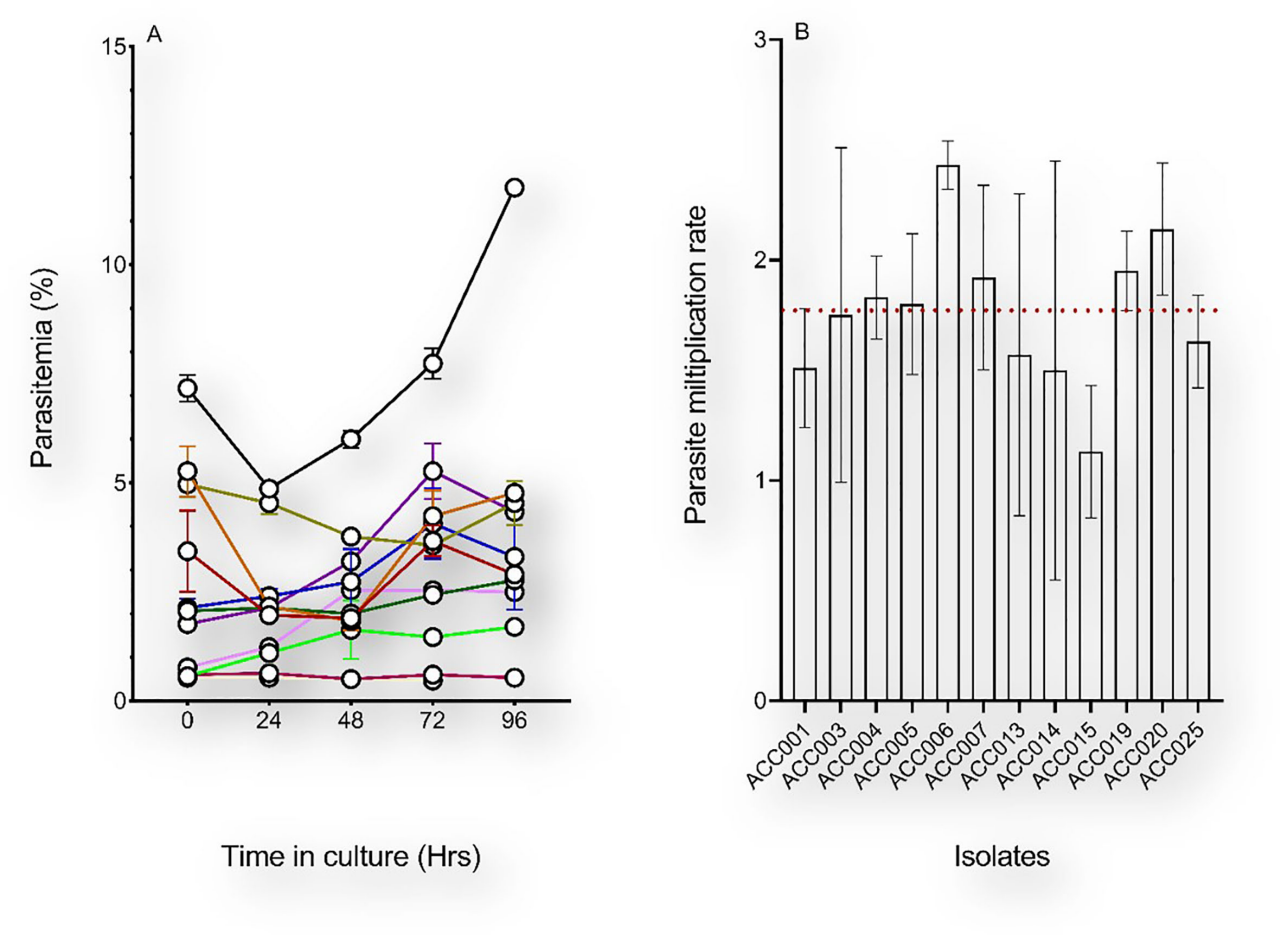
Early *in vitro* adaptation of *P. falciparum* clinical isolates. **(A)** The initial parasitemia (at H0) of each sample was recorded upon arrival from the field and the parasitemia of *ex vivo*-cultured isolates were monitored for 96 hours. The medium in the culture flasks was changed daily and supplemented with fresh erythrocytes only after 96 hours *in vitro*. **(B)** Parasite multiplication rates of successfully culture-adapted parasites following culture dilution with fresh erythrocytes. Depicted on the graph are the mean and standard errors of the PMRs of parasites with successful growth tests over a period of 28 days *in vitro*, and the red dotted line represents the median PMR (1.77).

Samples used in this study were collected from Ghanaian children with uncomplicated malaria visiting the LEKMA hospital in Accra. To monitor the *in vitro* growth patterns of freshly collected *P. falciparum* clinical isolates, parasites were initially allowed to grow in the patient-derived erythrocytes for a minimum period of 96 hours after which freshly washed O^+^ erythrocytes (from a single donor) (Thiam et al., 2021) were added and growth tests were performed for isolates that successfully grew. Of the 25 isolates used in this study, 19 (76%) yielded detectable parasitemia 48 hours following *in vitro* adaptation. As measured by flow cytometry, *P. falciparum* clinical isolates showed different growth patterns during the first 96 hours of *in vitro* adaptation (**Figure 2A**). Monitoring of the PMR during these first two *in vitro* cycles revealed that most of the isolates had less than two-fold increase in parasitemia from one cycle to another. Isolates with successful growth patterns were further diluted with fresh erythrocytes and the parasitemia was used to assess the parasite multiplication rate through successive growth tests for a maximum period of twelve successive replication cycles. Overall, all isolates showed a minimum of 8 successful growth tests (66.67% success rate) with a median PMR of 1.77 (range 1.13 – 2.43) (**Figure 2B**).

### *P. falciparum* clinical isolates represent genotypically diverse parasite populations

Unlike laboratory strains, *P. falciparum* clinical isolates frequently harbour multiple parasite clones, which could modulate the parasite’s *in vitro* adaptability. Here, we assessed the presence of multiple parasite clones in each of the tested isolates using the highly polymorphic regions of *msp1* and *msp2* genes. All 25 isolates were shown to be polyclonal and the number of alleles detected were 74 and 44 for *msp1* and *msp2*, respectively (**Table 1**). For *msp1*, the allele frequencies were 36.47% (27/74), 32.43% (24/74) and 31.10% (23/74) for K1, MAD20 and RO33, respectively, while those for the *msp2* allelic families were 54.54% (24/44) and 45.46% (20/44) for 3D7 and FC27, respectively. The prevalence of multiple infections was respectively 16 and 14 for *msp1* and *msp2*, while the multiplicity of infections was 2.6 and 1.76, respectively for the two genes (**Table 1**). Besides, the H_E_ was 0.35 and 0.52 for *msp1* and *msp2*, respectively.

**Table 1:** *Plasmodium falciparum* genetic diversity and *msp-1* and *msp-2* allelic diversities.

Allelic type	N (%)	Allelic type	N (%)
msp-1		msp-2	
K1	22 (15.83)	3D7	22 (40.74)
MAD20	21 (15.11)	FC27	18 (33.33)
RO33	23 (16.57)	3D7/FC27	14 (24.93)
K1/MAD20	18 (12.95)		
K1/RO33	20 (14.39)		
MAD20/RO33	19 (13.67)		
K1/MAD20/RO33	16 (11.51)		
Total combination	139 (100)		54 (100)
Total k1	27 (36.47)	Total 3D7	24 (54.54)
Total MAD20	24 (32.43)	Total FC27	20 (45.46)
Total RO33	23 (31.10)		
Total Alleles	74		44
MI	16 (64)		14 (76)
MOI	2.64		1.76

MI, Prevalence of multiple-infections, MOI, Multiplicity of infection.

### Short-term culture adapted isolates harbour lower number of parasite clones

**Figure 3:**
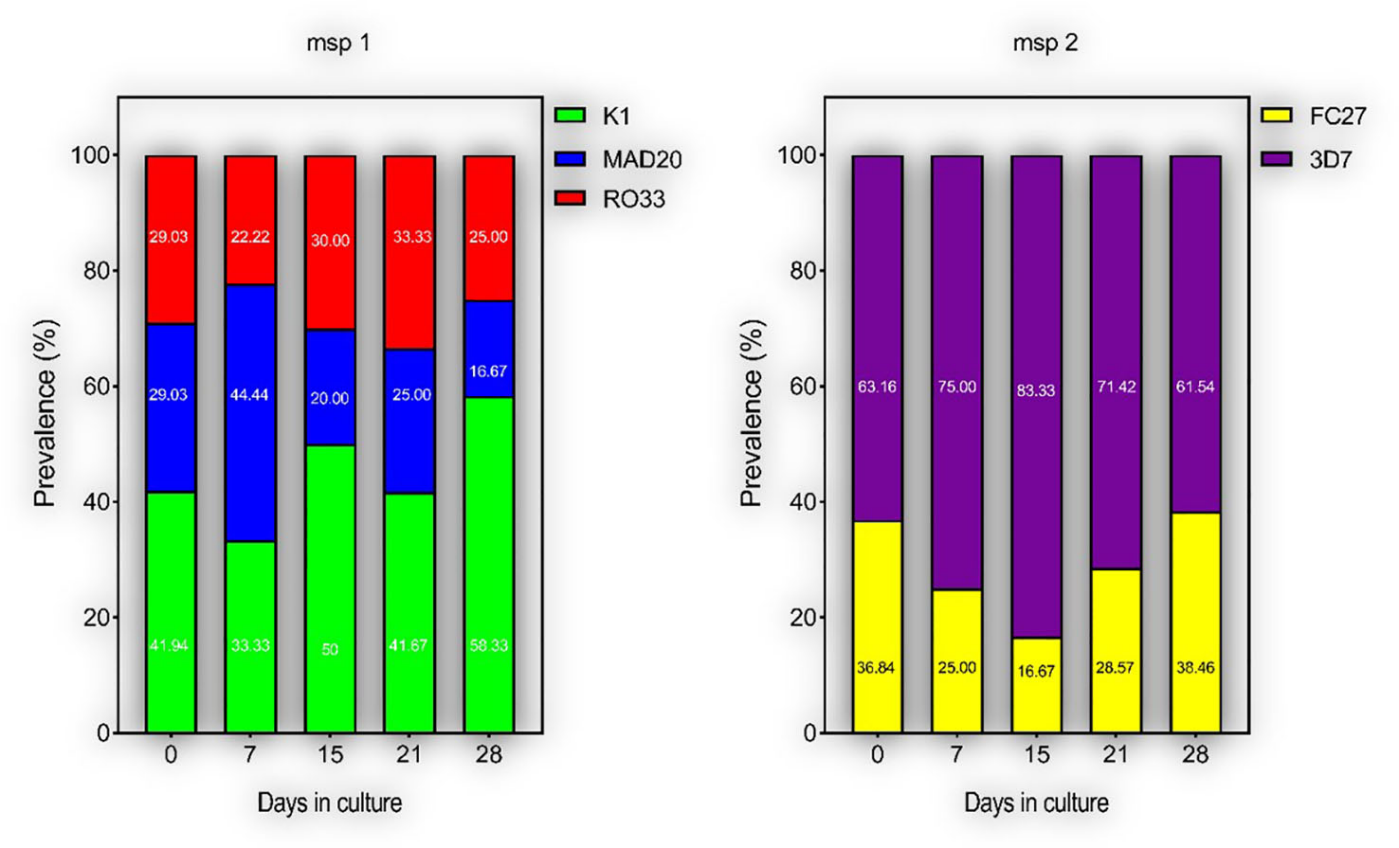
Proportions of different parasite clones during culture-adaptation. Parasites were all genotyped at Day 0 upon collection and successfully culture-adapted ones were further genotyped at Days 7, 15, 21 and 28-post adaptation. Represented are the proportions of individual alleles for the *msp 1* and *2* genes.

To measure the effect of short-term culture adaptation on the parasites’ genotypes, ten of the culture-adapted isolates were genotyped at days 7, 15, 21 and 28 post-adaptation using the *msp1* and *2* allelic families. Overall, there was little variation in the proportion of allelic families across the different time points (**Figure 3**). However, our analysis showed a reduction in the number of clones per isolate at day 28 as compared to day 0 (**Table 2**). The maximum number of clones per isolate was reduced from 4 to 1 for the *msp1* gene, while that of *msp2* was reduced from 2 to 1 (**Table 2**). Of all *msp1* allelic families, K1 was the most predominant at both day 0 and day 28, with a percentage of 41.94% and 58.33%, respectively (**Table 2**). MAD20 and RO33, which were present at the same proportion at day 0 (29.03%, each), represented respectively 16.67% and 25.00% of the total number of alleles (**Table 2**). For *msp2*, there were little changes in the proportions of the respective allelic families, with 3D7 being the most predominant allele representing 63.16% and 61.55% at day 0 and 28, respectively (**Table 2**). Out of the eight isolates that harboured the MAD20 allelic family at day 0, only two were detected with a copy of the allele at day 28, while the RO33 allele which was initially present in nine isolates at day 0, was detected in only three isolates at day 28. K1, initially detected in all ten isolates, was still present in seven of them at day 28. Of all three *msp1* allelic families, only K1 and RO33 were simultaneously detected in the same isolates at day 28, while MAD20 was only detected as single infections (**Table 2**).

**Table 2:** Comparative analysis of *P.falciparum* genetic diversity prior and following short-term culture adaptation.

		MSP-1			MSP-2		MSP-1			MSP-2	
		Day 0					Day 28				
**Number of clones per isolate**	Isolates	K1	MAD20	RO33	3D7	FC27	K1	MAD20	RO33	3D7	FC27
	ACC001	2	1	1	1	1	1	0	0	0	1
	ACC003	2	1	1	2	1	1	0	1	1	0
	ACC004	1	2	1	1	1	0	1	0	0	1
	ACC005	1	1	1	1	1	0	1	0	1	0
	ACC006	2	1	0	2	1	1	0	0	1	1
	ACC013	1	1	1	1	1	1	0	1	1	1
	ACC014	1	1	1	1	1	1	0	0	1	1
	ACC015	1	1	1	1	0	1	0	0	1	0
	ACC018	1	0	1	1	0	0	0	1	1	0
	ACC019	1	0	1	1	0	1	0	0	1	0
**Total**		13	9	9	12	7	7	2	3	8	5
**Percentage**		41.94	29.03	29.03	63.16	36.84	58.33	16.67	25.00	61.54	38.46

For the *msp2* gene, out of the seven isolates harbouring the FC27 allelic family at day 0, only four persisted at day 28, while the 3D7 allelic family was initially present in all ten isolates at day 0 but was detected in eight of them at day 28. As for *msp1*, both allelic families of *msp2* were present in three isolates as co-infections, while two and four isolates presented single infections of FC27 and 3D7, respectively at day 28. However, there was no specific dominant combination of *msp1* and *msp2* detected in our isolates at day 28. Moreover, aside those detected at day 0, there were no newly detected alleles in our isolates at day 28, therefore suggesting the absence of detectable cross-contamination during culture adaptation.

### Differences in thawing protocols does not affect early *in vitro* culture adaptation of short-term cryopreserved *P. falciparum* clinical isolates

**Figure 4:**
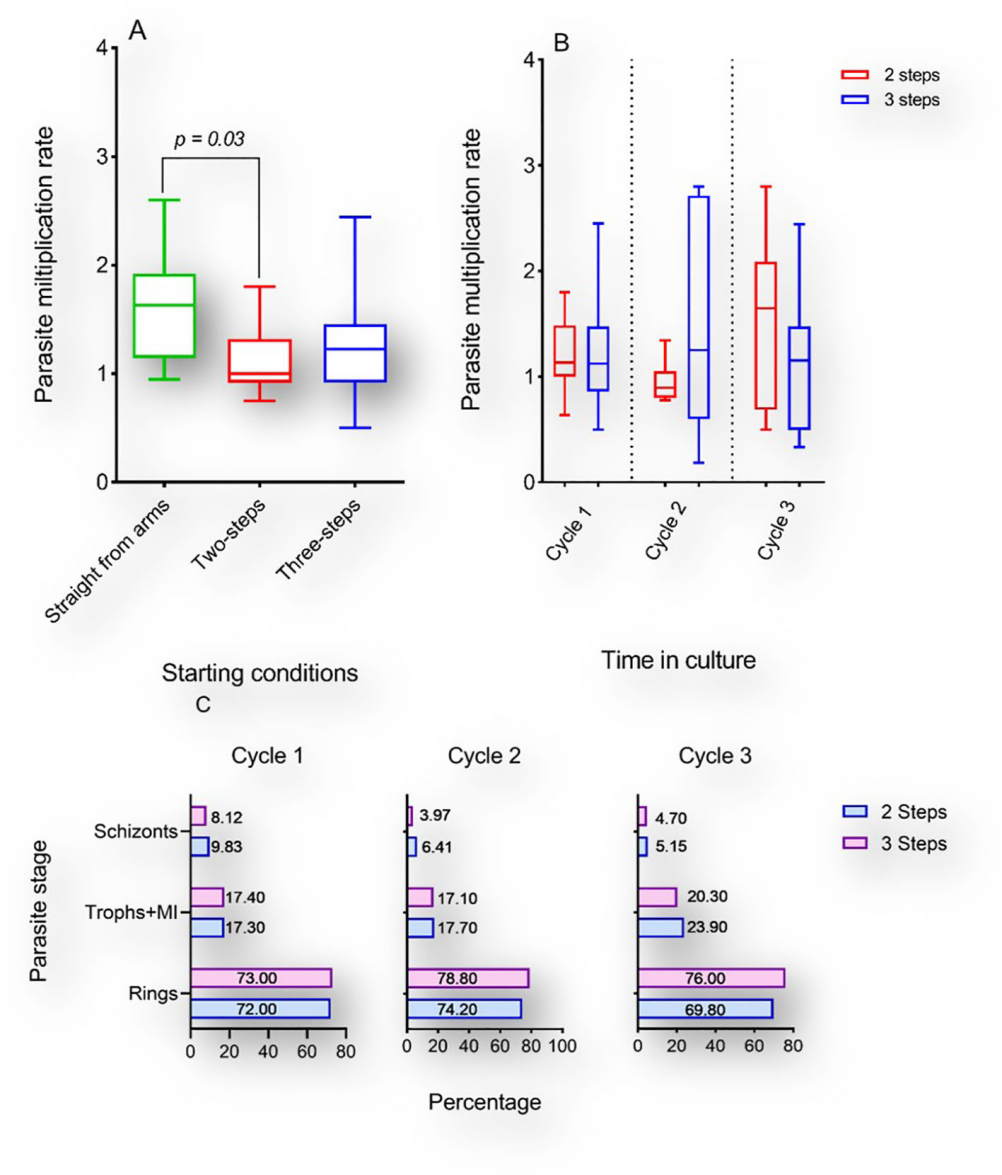
Multiplication rates of P. *falciparum* clinical isolates before and after cryopreservation. A-B: Box and whiskers plots showing the PMR of fresh cultured *P. falciparum* clinical isolates (green) or thawed with a two-step (red) or three-step (blue) protocol following cryopreservation. Kruskal Wallis test was conducted to compare the PMR of fresh versus cryopreserved isolates after three *in vitro* replicative cycles **(A)** or to compare the PMR of isolates thawed with different protocols after each replicative cycle **(B)**. Represented in **(C)** are the proportions of different parasites stages after 1, 2 and 3 cycles following thawing with either protocol.

Culture-adaptation of *P. falciparum* clinical isolates has been reported as more labor-intensive than immediate *ex vivo* processing. Moreover, there are earlier reports of *P. falciparum* clonal selection during *in vitro* adaptation (Chen et al., 2014). To ascertain the effect of cryopreservation on *P. falciparum* early *in vitro* adaptation, we compared the PMR of short-term cryopreserved clinical isolates to that of their freshly cultured isogenic counterparts. Two vials of the same isolate were simultaneously revived using two distinct NaCl-based thawing protocols. Parasitemia were adjusted to 0.5-1% using erythrocytes from a single donor and the PMR was monitored during the first three asexual replicative cycles. Successful monitoring of the PMR of isolates prior to and following cryopreservation (one-year interval) revealed differences in PMR between cryopreserved isolates and the matched freshly culture-adapted counterparts. The median PMR was 1.62 for fresh isolates while that of cryopreserved isolates was 1.12 and 1.27 following two-step and three-step thawing, respectively (**Figure 4A**). However, the difference in PMR was only significant when comparing fresh isolates and cryopreserved parasites thawed using a two-step protocol (P = 0.03; **Figure 4A**), while no significant difference was observed between isogenic isolates thawed using distinct protocols (**Figure 4A, B**). Flow cytometric analysis of the DNA content of revived cryopreserved parasites revealed different fluorescence peaks following Hoechst 33342 staining suggesting the presence of different parasite stages after 48-, 96- and 144-hours post-incubation. However, no significant difference was observed in the proportions of the different parasite stages when comparing the parasites’ growth after thawing using different protocols (**Figure 4C**).

### Short-term culture adaptation has minimal effect on *P. falciparum* invasion phenotype

**Figure 5:**
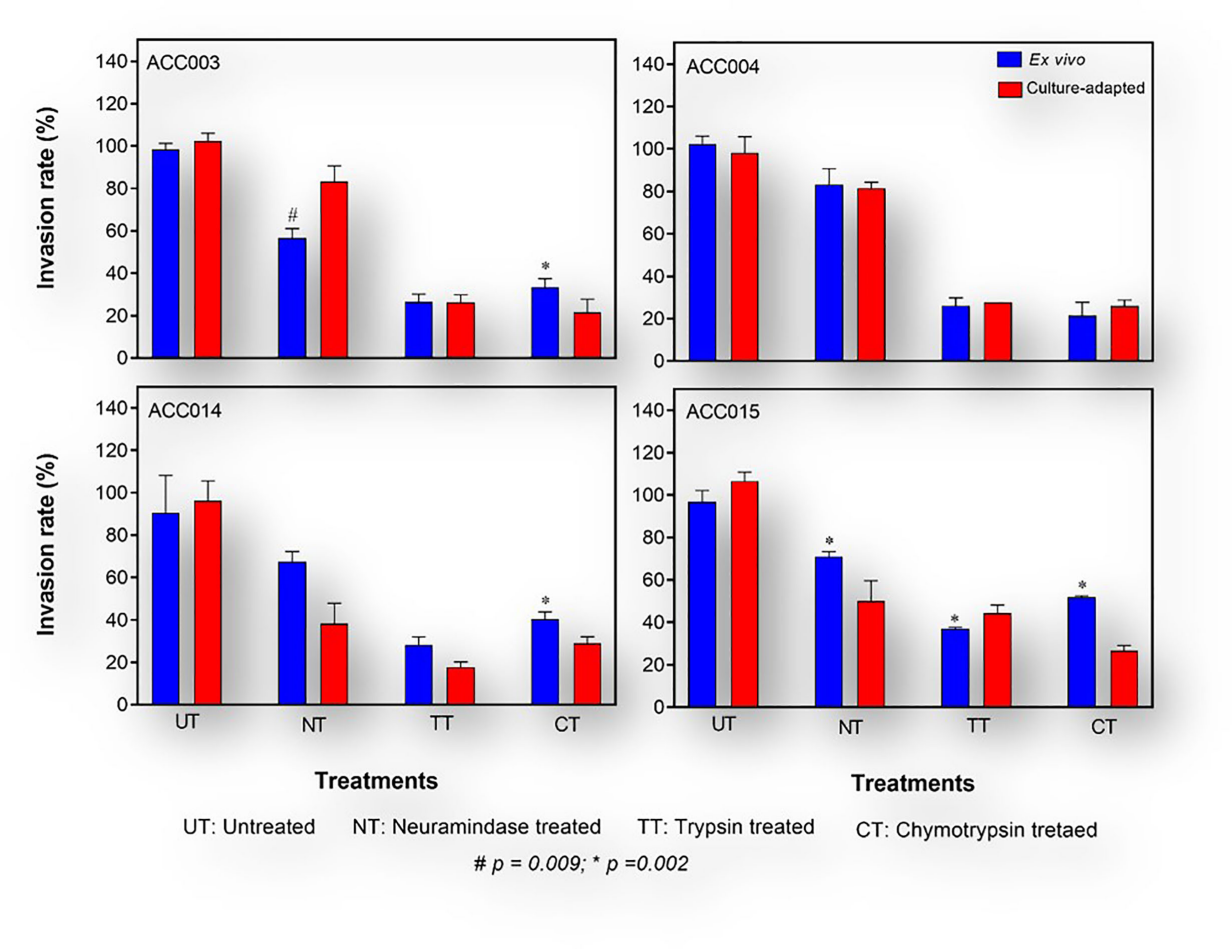
Invasion phenotypes of *P.falciparum* clinical isolates before and after short-term culture adaptation. The *ex vivo* phenotype of freshly collected isolates (blue bars) obtained during the first asexual replicative cycle upon arrival at the laboratory was compared to that obtained after a short-term *in vitro* adaptation of about 28 days (red bars). The Mann Whitney U test was used to assess the differences in invasion efficiency between the different time points.

*P. falciparum* invasion phenotyping has mostly been conducted following short-term culture-adaptation (Okoyeh et al., 1999; Lobo et al., 2004; Nery et al., 2006; Lopez-Perez et al., 2012). Invasion phenotypes are classified as either resistant (r) or susceptible (s) to the different enzyme treatment of the erythrocytes with invasion efficiency set at 50%. To this, we assessed the effect of short-term culture adaptation on *P. falciparum* invasion phenotype in the different enzyme treated erythrocytes. To allow for easy inference, we compared the *ex vivo* invasion phenotype of four isolates when the samples were collected to that obtained after 28 days in culture to see if there are changes. Of the four isolates tested, only one (ACC015) had a significant change in the sensitivity to enzyme treatment following culture adaptation (**Figure 5**), while significant changes were observed in ACC003 and ACC014 following treatment with neuraminidase and trypsin, respectively.

Moreover, changes in invasion profile, defined as the combination of sensitivity to the three enzymes, was observed in two isolates (ACC014: NrTsCs → NsTsCs and ACC015: NrTsCr → NrTsCs), where N: neuraminidase, T: trypsin, C: chymotrypsin, s: sensitive and r: resistant; while → depicts the changes in profile). Interestingly, although all three msp1 alleles were initially present in these two isolates, only K1 persisted at Day 28-post adaptation, while both the 3D7 and FC27 alleles of msp2 persisted throughout the 28-day period post adaptation.

### Cryopreservation has minimal effect on *P. falciparum* invasion phenotype

**Figure 6:**
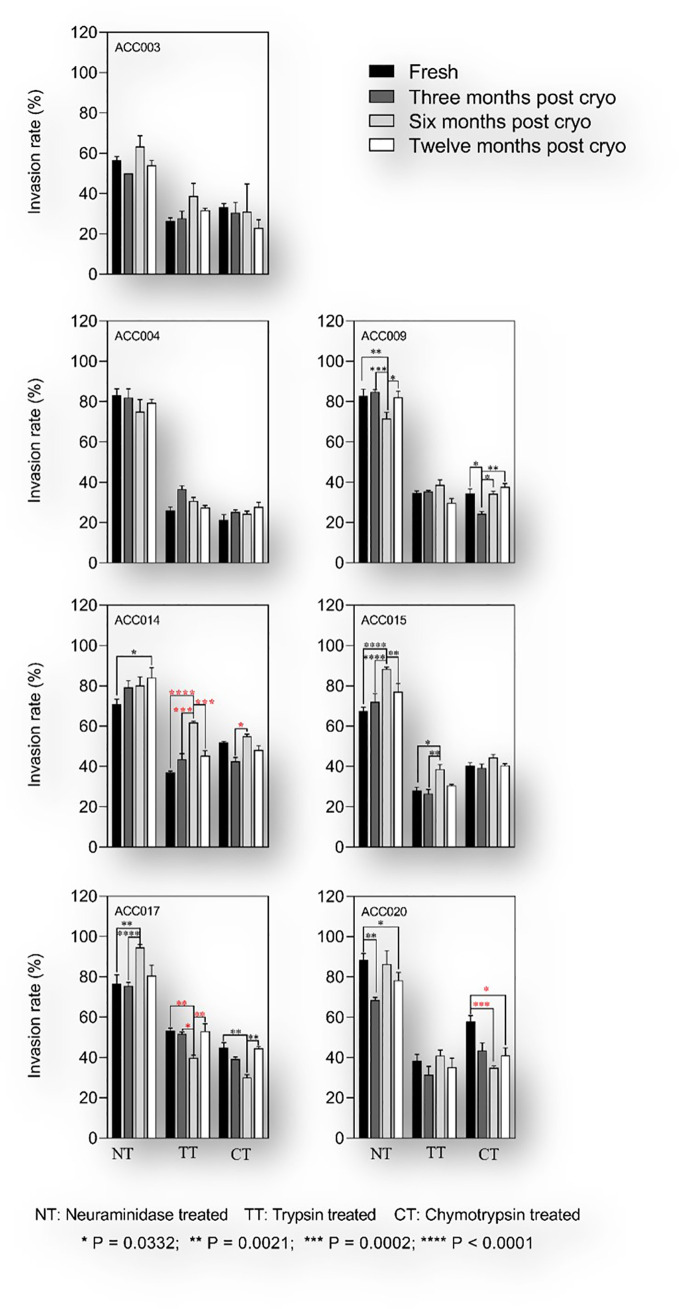
Invasion phenotypes of *P.falciparum* clinical isolates before and after short-term cryopreservation. The assays were set up between 24- and 36-hours following sample processing and the parasites were incubated for another 18 to 24 hours prior to flow cytometric analysis. For each isolate, the invasion phenotype of freshly culture adapted parasites (black bars) was compared to that obtained after three months (dark grey bars), six months (light grey bars) and twelve months (white bars) post cryopreservation. Kruskal Wallis was conducted to test for statistical differences in invasion efficiency of fresh versus cryopreserved isolates. Red stars denote significant differences associated with changes in invasion profile of a given treatment and black stars depict significant differences with no changes in the invasion profile.

Cryopreserved isolates were thawed at different time intervals (from 3 to 12 months after cryopreservation) and assayed for their ability to invade enzyme-treated erythrocytes as compared to their fresh uncultured counterparts. Given the challenges associated with the parasites’ *in vitro* adaptability during the first rounds of asexual replication, and to avoid clonal selection following long-term culture adaptation, all assays were set during the first two replicative cycles. Of the 25 isolates collected in this study, seven had enough numbers of cryopreserved vials (six vials) to be thawed at all the time-points and successfully phenotyped over the course of one-year post cryopreservation. All isolates showed a sialic acid independent phenotype with the invasion of neuraminidase treated erythrocytes greater than 50% relative to that of untreated control erythrocytes (**Figure 6**). Overall, there was no significant change in sialic acid dependency in the parasites after cryopreservation relative to fresh uncultured isolates (**Figure 6**). The most common invasion profile was neuraminidase resistant, trypsin sensitive and chymotrypsin sensitive (NrTsCs). However, three out of the seven isolates showed changes in invasion profiles in trypsin and chymotrypsin treated erythrocytes following cryopreservation with the apparition of three novel phenotypes (NrTrCs, NrTsCr and NrTrCr) after six months post cryopreservation (**Figure 6**). Nevertheless, none of the novel phenotypes persisted after twelve months post cryopreservation, suggesting a technical or random effect associated with these changes.

## Discussion

The phenotypic diversity of *P. falciparum* clinical isolates has widely been reported in the last two decades (Ahouidi et al., 2016). However, conducting such assays with uncultured clinical isolates has been precluded by the lack of appropriate laboratory settings in remote areas where the highest malaria burden occurs. Consequently, the majority of the pioneering works were conducted in cryopreserved *P. falciparum* clinical isolates and in some cases after short-term culture adaptation following parasite thawing (Okoyeh et al., 1999; Lobo et al., 2004; Nery et al., 2006; Lopez-Perez et al., 2012), although the effect of such procedures in the parasite’s invasion phenotype has never been reported.

In this study, we investigated the effect of cryopreservation and different thawing protocols on *P. falciparum in vitro* adaptation and invasion phenotyping assays. We showed that *P. falciparum* clinical isolates show specific growth patterns during the early *in vitro* culture adaptation, while most of the isolates did not recover following culture dilution with fresh erythrocytes. Previous studies have reported such findings, mainly attributed to unreported antimalarial drug use prior to presentation at hospital (Baum et al., 2003).

Given that *P. falciparum in vitro* adaptation could also be influenced by the number of parasite clones present in a given isolate, our genotyping analysis revealed that all *P. falciparum* clinical isolates used in this study presented multiple parasite clones, with an overall MOI of 4.4, which is slightly higher than previously reported in Burkina Faso (Sondo et al., 2019), Ghana (Duah et al., 2016), Republique of Congo (Singana et al., 2019), Nigeria and Senegal (Oboh1 et al., 2017). However, the predominance of K1 and 3D7 allelic families of msp1 and msp2 reported here is in agreement with previous reports from Burkina Faso (Sondo et al., 2019; Sondo et al., 2020), Nigeria and Senegal (Oboh1 et al., 2017) Burkina Faso (Oboh1 et al., 2017; Sondo et al., 2019) (Kobbe et al., 2006), but contrary to studies from Uganda and Sudan which reported the predominance of RO33 and FC27, respectively for the two genes (Peyerl-Hoffmann et al., 2001; Mohammed et al., 2015).

In this study, the median PMR of short-term culture adapted isolates was 1.77, consistent with previous reports (Lantos et al., 2009), however, there was no relationship between the observed PMR and the number of parasite clones per isolate. Furthermore, of the 19 culture-adapted isolates tested in this study, 10 were successfully genotyped at day 28 post culture inoculation and our data show an apparent clonal selection following short-term culture adaptation. This is in agreement with previous data that also reported culture adaptation of *P. falciparum* clinical isolates as a modulator of the parasite’s susceptibility to a wide range of drugs as compared to their fresh uncultured counterparts (Chaorattanakawee et al., 2015).

Our data also show the persistence of K1 at day 28 in almost all isolates that harboured this allelic family on day 0, while MAD20 prevalence significantly decreased at day 28. Furthermore, MAD20 was outgrown by K1 in almost all isolates harbouring mixed infections (8/10 isolates), consistent with reports by Sondo and colleagues where MAD20 was outcompeted by K1 in natural malaria infections (Sondo et al., 2019).

For some of the isolates in this study, the invasion phenotype following culture adaptation was different from the parasites’ *ex vivo* phenotype. This, therefore, suggests a possible effect of short-term culture adaption and/or clonal selection on the parasite invasion phenotype. However, this could also result from technical variations during invasion assay set up, and given the small number of isolates tested for this experiment (only four isolates), there is a need for further confirmation with a larger number of isolates and possibly a longer culture adaptation time.

The present study also investigated the effect of cryopreservation and thawing protocols in the parasites’ early *in vitro* adaptation and invasion phenotype as compared to their freshly cultured isogenic counterparts. Our data show that cryopreserved *P. falciparum* clinical isolates used in this study have a lower multiplication rate during the first *in vitro* cycles as compared to their fresh isogenic counterparts. However, this difference in PMR was only significant when freshly cultured isolates were compared to those revived using a two-step NaCl protocol. This could be because of a simple artefact during culture adaptation or due to the fitness of the different parasite clones in each isolate. It is therefore possible that the parasite-induced stress during the thawing process will accentuate the low fitness of certain clones, while this effect could be minimal when parasites are thawed using a three-step protocol.

Another potential factor that could affect the early *in vitro* adaptation of revived cryopreserved isolates is the addition of fresh erythrocytes following parasites growth. Given the difference between the freshly added erythrocytes and the initial patient-derived erythrocytes, there is a possibility that the lower multiplication rate observed post-cryopreservation is solely due to the parasite adaptation to the new erythrocytes. However, given the observed differences in the PMR post cryopreservation, and to minimize the effect of clonal selection, invasion phenotyping assays were only performed in isolates that yielded appropriate parasitemia during the first *in vitro* replicative cycles.

As a result, our data show that most of the isolates assayed following cryopreservation maintained a relatively stable invasion phenotype regardless of the length of cryopreservation or the thawing protocol used for the revival of the parasites. To our knowledge, this is the first study to investigate the effect of short-term culture adaptation and cryopreservation on *P. falciparum* invasion phenotype using clinical isolates from the same isogenic backgrounds. Altogether, these results suggest that short-term culture adaptation could influence the invasion phenotype of *P. falciparum* clinical isolates due to clonal selection during *in vitro* culturing, but this needs further confirmatory studies with a larger number of isolates.

## Data availability statement

The original contributions presented in the study are included in the article/supplementary material. Further inquiries can be directed to the corresponding authors.

## Ethics statement

The studies involving human participants were reviewed and approved by Institutional Review Board of the Noguchi Memorial Institute for Medical Research, University of Ghana (IRB00001276) and the Ghana Health Service Ethical Review Committee (GHC-ERC: 005/12/2017). Written informed consent to participate in this study was provided by the participants’ legal guardian/next of kin.

## Author contributions

The authors want to indicate that data presented in this manuscript forms part of thesis submitted to the University of Ghana in partial fulfillment for the award of Ph.D. to LT 28. LT, YA and GA conceived the study; LT and FA performed the experiments LT, FA, MN, YA and GA analyzed the data and drafted the manuscript; YA, GA, and MN supervised the study. All authors critically reviewed and edited the manuscript.

## Funding

This work was supported by funds from a World Bank African Centres of Excellence grant (ACE02-WACCBIP: Awandare) and a DELTAS Africa grant (DEL-15-007: Awandare). LT was supported by WACCBIP-World Bank ACE PhD fellowships, respectively, FA was supported by the National Institute for Health Research (NIHR) Global Health Research program 16/136/33, using aid from the UK Government while YA was supported by a WACCBIP-DELTAS postdoctoral fellowship. The DELTAS Africa Initiative is an independent funding scheme of the African Academy of Sciences (AAS)’s Alliance for Accelerating Excellence in Science in Africa (AESA) and supported by the New Partnership for Africa’s Development Planning and Coordinating Agency (NEPAD Agency) with funding from the Wellcome Trust (107755/Z/15/Z: Awandare) and the UK government. The views expressed in this publication are those of the author(s) and not necessarily those of AAS, NEPAD Agency, Wellcome Trust or the UK government.

## Acknowledgments

This work is part of the assay standardization efforts of the West African merozoite invasion network (WAMIN) consortium, and we are grateful to members for contributing ideas to this work.

## Conflict of interest

The authors declare that the research was conducted in the absence of any commercial or financial relationships that could be construed as a potential conflict of interest.

## Publisher’s note

All claims expressed in this article are solely those of the authors and do not necessarily represent those of their affiliated organizations, or those of the publisher, the editors and the reviewers. Any product that may be evaluated in this article, or claim that may be made by its manufacturer, is not guaranteed or endorsed by the publisher.
